# Timescale mediates the effects of environmental controls on water temperature in mid- to low-order streams

**DOI:** 10.1038/s41598-022-16318-9

**Published:** 2022-07-18

**Authors:** Jorge García Molinos, Ishiyama Nobuo, Masanao Sueyoshi, Futoshi Nakamura

**Affiliations:** 1 Arctic Research Center, Hokkaido University, Sapporo, Japan; 2 Hokkaido Research Organization, Forestry Research Institute, Bibai, Japan; 3 Aqua Restoration Research Center, Public Works Research Institute, Gifu, Japan; 4 Research Faculty of Agriculture, Hokkaido University, Sapporo, Japan; 5 Present Address: Biodiversity Division, National Institute for Environmental Studies, Ibaraki, Japan

**Keywords:** Freshwater ecology, Environmental sciences, Limnology

## Abstract

Adequate management and conservation of instream thermal habitats requires an understanding of the control that different landscape features exert on water temperatures. Previous studies have extensively explored the influence of spatial scale on these relationships. However, the effect of temporal scale remains poorly understood. Here, we use paired air–water mean daily and monthly summer temperatures collected over four years from 130 monitoring stations in Japanese mid- to low-order streams to investigate whether perceived effects of different environmental controls on water temperature are dependent on the timescale of the temperature data, and whether those dependencies are related to the spatial scale at which these controls operate. We found a clear pattern for the significant cooling effect, high relative importance and strong dominance exerted by the riparian forest cover on daily temperatures at the reach scale becoming dampened by concomitant increases associated to the proportion of volcanic geology on monthly temperatures at the catchment scale. These results highlight the importance of contextualizing the effects of environmental controls on water temperatures to the timescale of the analysis. Such dependencies are particularly important for the management and conservation of instream thermal habitats in a rapidly warming world.

## Introduction

Increasing thermal stress resulting from human disturbances poses a risk to freshwater biota, given the control of temperature on physiological processes^1^, prompting them to respond *in situ* by exploiting local thermal refugia^2^ or by relocating to colder tributaries and upstream reaches^3^. As a result, characterizing instream thermal habitats and their controlling factors at both local and broader, basin-wide scales is becoming increasingly important for the integrated management and conservation of fluvial ecosystems and associated biota^4,5^.

Multiple climatological, hydrological, morphological and geological factors influence stream water temperature across spatial scales through their effect on heat exchange processes occurring at the stream surface and streambed interfaces^6^. A growing body of literature demonstrates how spatial heterogeneity in the characteristics of river networks and their catchments can shape localized responses of stream temperature sensitivity, which can vary significantly across space even in regions that are geographically close and climatically similar^7–9^. This makes the extrapolation of simple air–water temperature relationships beyond monitored sites across river networks problematic. Yet predictions of water temperature are needed at scales meaningful to management (i.e., catchment scale). Recent modelling efforts try to circumvent this issue by explicitly incorporating the effect of landscape and geomorphological characteristics into the prediction of stream water temperatures^10–12^.

Thermal regimes are also geographically distinct and scale dependent in the time domain^13^. Previous studies have shown that air-water temperature relationships vary significantly with the temporal resolution of the data, where higher slopes and lower intercepts are usually associated when regressing water on air temperature at increasing timescales^6^. However, much less is known about how temporal scale can modify perceived effects of other environmental controls on stream water temperatures over and above the effect of air temperatures. Using spatial stream network models, Steel et al.^14^ found that relationships between landscape predictors and water temperature in the Snoqualmie River changed among temperature metrics defined for different temporal windows and timescales. For example, while increasing elevation correlated strongly with decreasing summer mean temperatures, this relationship was reversed for mean daily thermal range and lost for temperature variability defined for multiple daily and hourly timescales. These results are important because the biological and ecological relevance of temperature metrics used to represent stream thermal regimes vary with timescale. Short-term average temperatures and thermal variability are arguably more relevant to physiological processes and related lethal and sublethal responses in ectotherms^15^, while temperature aggregated over larger timescales is useful for assessing broader ecological patterns and overall distributions of species across geographical ranges^16,17^. Management of instream thermal environments and associated biodiversity requires, therefore, a better understanding of the dependencies between environmental controls and stream temperatures across timescales^18^.

**Figure 1:**
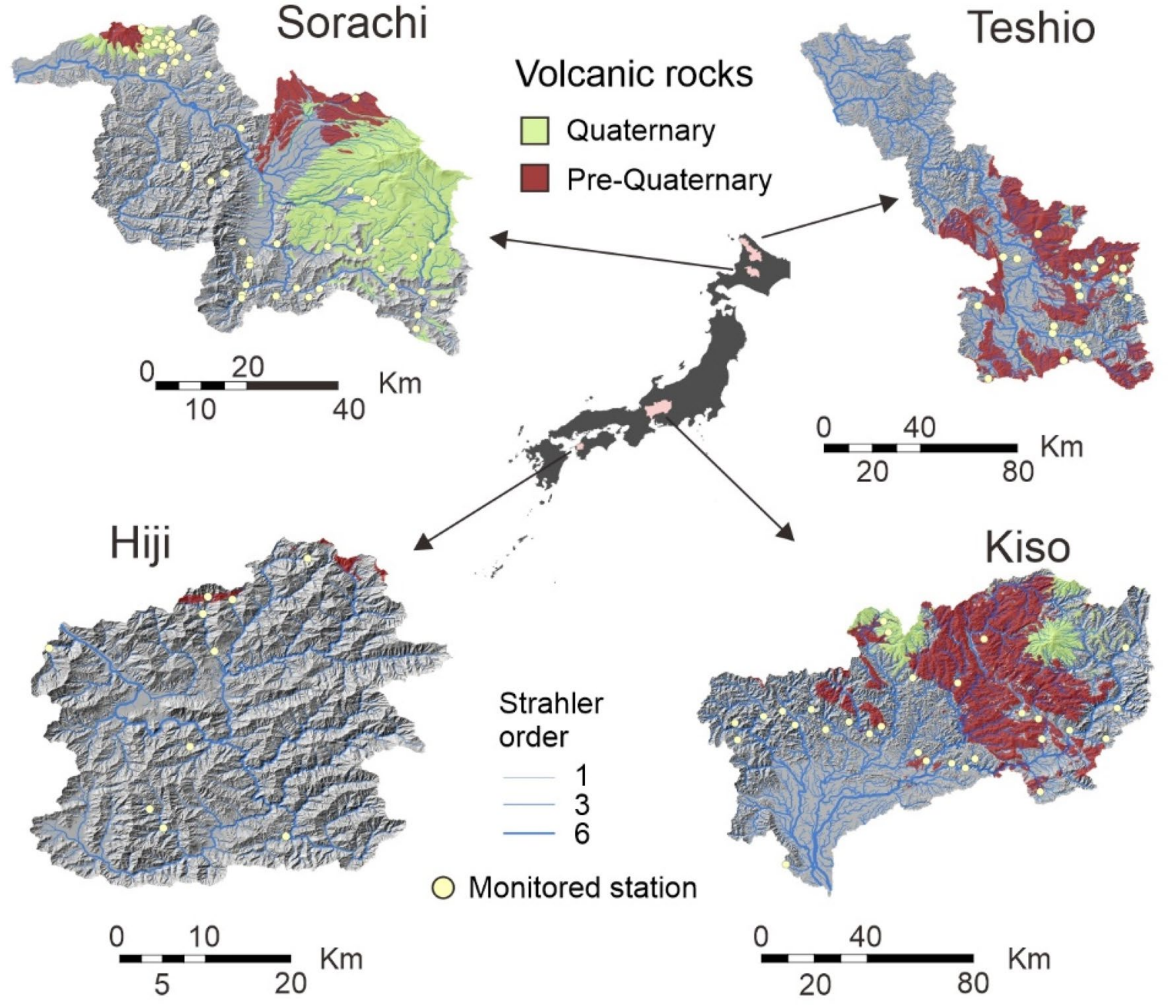
Location of the four study catchments and the paired temperature stations (*n* = 130) set to monitor air and water temperatures. The width of the river lines is proportional to their Strahler order. The distribution of volcanic formations grouped by age is shown in the background. Maps created with ArcGIS Desktop 10.7.1 (https://www.esri.com).

Here we use summertime paired air-water temperatures, collected from 130 monitoring sites in Japanese mid- to low-order streams, i.e. from headwaters to middle reaches, to explore the temporal dependency of the effects of local- and catchment-scale environmental predictors on stream water temperatures after controlling for the effects of air temperature. Specifically, we use linear mixed-effect models and dominance analysis^19^ to identify changes in the relative importance of individual predictors and their dominance relationships. We focus our analysis on the control exerted by land use and solid geology, at the catchment scale, and the riparian cover and channel elevation, at the reach scale, because these are all predictors frequently used in, and of demonstrated importance for, the prediction of stream water temperatures^6^. Specifically, we focus on the effect of volcanic rocks, abundant in our study catchments (Fig. 1), given their strong contribution towards cold groundwater discharge relative to other bedrock types^20^. We further make a distinction based on geological age, as young volcanic rocks with high porosity and permeability are frequently associated to more prolific unconfined acquirers relative to older formations^20^. These considerations are supported by results from previous related studies conducted in Japanese streams^21,22^, including our own work in the study catchments^23^.

## Material and methods

### Study catchments and temperature monitoring networks

We conducted our study at four catchments in Japan (Fig. 1): the Sorachi and the Teshio Rivers in Hokkaido, the Hiji River in Shikoku, and the Kiso River in Honshu. Upper sections of these catchments are mostly covered by deciduous and evergreen mature forest with a geology dominated by metamorphic, plutonic and extrusive volcanic rocks of mixed ages (Supplementary Figs. S1, S2). Agricultural, paddy field and urban land uses dominate in the fluvial valleys and lower sections of the catchments with geologies mostly comprising accretionary complexes, sedimentary rocks and unconsolidated sediments. Extrusive volcanic formations, on which we focus this study, are mainly represented by felsic (dacite and rhyolite), mafic (basalt and andesite) and, in much lower proportions, volcanic debris and pyroclastic flow (pumice and volcanic ash) rocks (Fig. S2).

This study is based on data collected during the summer (June-September) between 2018 and 2021 from a total of 130 paired (air-water) temperature monitoring stations (Fig. 1). Stations were allocated across each river basin covering as much of their environmental gradients as possible subject to logistic and accessibility constraints. The monitored network covers only low- to mid-order streams (Strahler order 1 to 4) due to operational constraints and the high level of human alteration existing in the larger, non-wadable rivers of these four catchments.

Air and water temperatures were recorded at each site using Onset Hobo UA-001-64 (accuracy ± 0.53 °C) and Gemini TG-4100 (± 0.5 °C) temperature data loggers. Water loggers were installed in non-turbulent flowing sections of the stream housed in short sections of perforated PVC pipes to facilitate water exchange and shield them from direct sunlight. Air loggers on perforated PVC pipes were attached to nearby trees (~ 2 m high) in shaded areas by the stream bank^24,25^. Loggers were set to record temperatures every hour, which were then converted into mean daily and monthly temperatures using the 'timeAverage' function of the R package 'openair'^26^ at a 0.75 unit threshold. In other words, a given day or month was computed from the corresponding hourly data if at least 75% of the hours for that temporal unit were available. Otherwise the unit was taken as a missing observation. Visual inspection of the temperature series suggested some suspected periods of air exposure of water loggers in a few sites (Fig. S4), which were subsequently treated as missing observations. Missing observations occurred also sporadically due to issues such as faulty or lost loggers. Nonetheless, the proportion of missing observations within individual series was small across sites (1.79 ± 5.79%), resulting in a final total of 38,798 daily and 1324 monthly valid paired temperature observations with an average effective series duration by site of 10.19 ± 2.46 months (234.85 ± 144.23 days).

### Landscape variables

River networks and catchments were derived from the conditioned 10-m grid digital elevation model (DEM) form the Geospatial Information Authority of Japan (available from https://fgd.gsi.go.jp/download/) based on flow accumulating and minimum length thresholds of 5000 (~ 0.5 km^2^ minimum contributing catchment) and 100 cells (~ 1 km), respectively, using the Spatial Analyst Tools in ArcGIS 10.7.1. River network topology and different environmental layers were then queried at each monitored site to extract our set of predictor variables (Table 1).

**Table 1:** Description of catchment- and reach-scale environmental covariates measured at each monitored site including related hydrological and stream heat exchange processes (+ /− symbols indicate expected positive/negative relationships), and the source and format of the data sets used in this study to derive each variable.

Variable	Description	Relevance	Data source	Format
**I. Catchment scale**				
Cultivated area	Proportion of cultivated area (crop and paddy field uses) within contributing catchment	Surface run−off (+). Catchment responsiveness (+) and residence time (−)	ALOS/AVNIR−2 High Resolution Land Use/Cover map, Japan Aerospace Exploration Agency (JAXA)	10 m raster
Quaternary/pre−Quaternary volcanic rock	Proportion of formations classified as volcanic rocks within contributing catchment of Quaternary/pre−Quaternary origin	Hydrogeological processes. Groundwater contribution (+)	Seamless Digital Geological Map of Japan, Geological Survey of Japan, AIST	1:200,000 vector
**II. Riparian buffer**				
Reach elevation	Mean elevation of the 100 m stream reach centered on a site	River discharge (−), channel width (−), depth (−), and slope (+)	DEM Geospatial Information Authority of Japan	10 m raster
Riparian forest cover	Proportion of forest land use (evergreen, deciduous and bamboo forest) within upstream riparian buffer (120 m wide centered on the stream line and running 1 km upstream from each site). Where channel bifurcations where met, the buffer extended on all stems	Incident incoming solar radiation (−)	ALOS / AVNIR−2 High Resolution Land Use/Cover map, Japan Aerospace Exploration Agency (JAXA)	10 m raster

### Statistical models

We used linear mixed effects models (LMMs) for prediction of mean daily and monthly water temperatures following the general notation:

1 $$Tw_{i,j} = \alpha + \beta_{air} Ta_{i,j} + \beta X_{i,j} + \vartheta_{j,k} + h\left( {s_{j} ,\varphi_{i} } \right) + \epsilon_{i,j}$$

where *Tw*_*ij*_ and *Ta*_*ij*_ are the *i*-th observation of mean water and air temperatures for site *j*; *α* is the (grand) intercept; *βX*_*i,j*_ represents the corresponding linear combination of site-specific environmental covariates and their corresponding regression coefficients; *ϵ*_*i,j*_ are the observation-specific residuals; *υ*_*j,k*_ is a random effect that includes a random slope and intercept for air temperature at the site level, accounting for the different air–water temperature relationships observed across sites (Fig. S5); and $h({s}_{j},{\varphi }_{i})$ is a continuous temporal autoregressive (CAR) structure of order 1 at site level used in the daily model (see below) to account for temporal correlation in model residuals defined by *φ*, the correlation between two observations one unit of time apart, and *s* as the time covariate grouped by site^27^. We initially considered the inclusion of catchment as the highest level of a hierarchical nested random effect structure. However, we decided to include it as a fixed-effect categorical variable due to the limited sample size at the higher hierarchical level (i.e., 4 catchments). A bare minimum of five groups is usually considered necessary at the highest hierarchical random effect level to make meaningful estimates of the variation among random effects (group-level) in hierarchical mixed-effects models^28,29^.

Examination of the normalized residuals by site for the daily model showed a clear, consistent pattern of a gradually decaying autocorrelation function and a partial autocorrelation function with a strong significant correlation (~ 0.8–0.9) at lag 1 dropping sharply below the significance threshold thereafter (Fig. S6). Therefore, we included a continuous autoregressive structure of lag 1 into the daily models, which flexibly allows for the existence of gaps in the time series of records^27^. These temporal autocorrelation patterns were absent in the monthly model (Fig. S6). Therefore, the temporal correlation structure was not added to the monthly model.

Despite the limited number of observations at the site level for the monthly model (10.19 ± 2.46 observations), simulation studies demonstrate that, when the number of groups is sufficiently large (i.e., 130 sites in our case), neither fixed nor random effects estimates are affected by small sample size within groups even in extreme situations^30,31^. Sample size at site level for the monthly model was hence considered to be sufficient to obtain unbiased estimates of the fixed effects for the monthly model at the population level (i.e., across sites), which constitute the focus of this study.

Model performance was assessed by mean of the conditional and marginal coefficients of determination R^2^^[,32,33^, representing respectively the total variance explained by the model (fixed and random components together) and the variance explained by the fixed effects. The root mean square error (RMSE) was also computed for each model as a measure of goodness-of-fit. All metrics were computed using the function 'model_performance' from the R package 'performance'^34^. Covariates were assessed for multicollinearity on the fitted models using variance inflation factors (VIFs) with a cut-off value of 10^35^. All predictors had VIFs well below that threshold in both models.

### Relative importance of predictors and dominance analysis

We assessed the relative importance of each predictor in the models using Dominance Analysis (DA)^19,36^. DA is a technique to rank-order the predictors in a model in terms of relative importance by comparing the additional contributions from each predictor to the variance explained by all subset models containing all possible combinations of the rest of the predictors. The additional contribution of a predictor to a given subset model is measured as the increase in the variance accounted for by that model after the addition of that predictor. Dominance can then be established among any pair of predictors present in the original model, where one predictor is said to dominate over another if its additional contribution is greater than that of the other predictor over all subset models not including any of the two predictors^19^. Azen and Budescu^36^ established three types of dominance that are hierarchically related. At the highest level, complete dominance of a predictor over another is established if the additional contribution of that predictor is consistently greater than the contribution of the other predictor over all possible subset models that do not include any of the two predictors. When this is not the case, weaker levels of dominance can still be established between pairs of predictors. Conditional dominance occurs when the average additional contribution of a predictor for all subset models within each model size is greater than that of another predictor. Finally, general dominance, representing the weakest level in the dominance hierarchy, occurs when the additional contribution of a predictor averaged across all model sizes is greater than that of the other predictor. Although several other related methods for assessing the relative importance of predictors based on an 'all subsets' approach exist^37^, their estimations focus only on average contributions over all predictor orderings (i.e., general dominance in DA). Therefore, DA provides the additional important insight given by the other relative importance measures (i.e., complete and conditional dominance)^38^.

Given our interest is in assessing the control of environmental predictors on water temperatures, we used a model fitting water temperatures to air temperatures plus the catchment effect as a baseline against which relative contributions of the environmental predictors were evaluated in terms of changes in marginal R^2^ while keeping the random component of the models invariant. This allowed us to assess predictor contribution to explain variability in water temperatures after accounting for the effect of air temperatures. All statistical computations were done in R version 3.6.3^39^.

## Results and discussion

The proportion of variance in water temperatures explained by the models increased with coarser temporal resolution with marginal and conditional R^2^ of 0.71/0.81 and 0.77/0.93 for mean daily and monthly temperatures, respectively. RMSEs (population-level) showed a reversed trend, with mean deviations of predicted water temperatures from observed water temperatures decreasing from 1.83 °C for mean daily temperatures to 1.41 °C for mean monthly temperatures. Models at both timescales improved the performance of the baseline model (R^2^_day_ = 0.5, RMSE_day_ = 2.39 °C, R^2^_month_ = 0.57, RMSE_month_ = 1.94 °C) significantly (*p* < 0.0001 ANOVA based on Maximum Likelihood model estimates), demonstrating the utility of the inclusion of environmental variables to capture variability in air–water temperature sensitivity across sites.

Air temperature had a highly significant positive effect on water temperature with a regression coefficient twice as big in the monthly compared to the daily model (Table 2). Steeper regression slopes and lower intercepts often result when increasing the timescale in simple water–air linear regression models because averaging over longer timescales decreases the variance in the data by reducing the effect of the time lag or transient response of stream water temperatures to changing air temperatures, typically operating on the order of hours to days^40,41^. The catchment effect was also significant in both models (Table 2); the southern catchments (Kiso and Hiji) having higher water temperatures on average relative to the northern (Sorachi and Teshio) catchments.

**Table 2:** Summary of the results for the linear mixed effects models fitting water temperature to air temperature and a suite of landscape and reach scale environmental covariates at the two timescales.

	Daily				Monthly			
Predictors	Coef	SE	*t*	*p*	Coef	SE	*t*	*p*
(Intercept)	14.425	0.439	32.837	** < 0.001**	6.647	0.625	10.63	** < 0.001**
Air Temperature	0.315	0.008	39.311	** < 0.001**	0.622	0.016	38.835	** < 0.001**
Catchment:Kiso (contrast Hiji)	−0.327	0.343	−0.954	0.342	−0.702	0.504	−1.392	0.166
Catchment:Sorachi	−3.922	0.305	−12.846	** < 0.001**	−1.987	0.455	−4.37	** < 0.001**
Catchment:Teshio	−3.626	0.368	−9.85	** < 0.001**	−1.544	0.494	−3.122	**0.002**
**Catchment scale**								
Cultivated area	0.033	0.015	2.272	**0.025**	0.043	0.015	2.869	**0.005**
Volcanic Quaternary	−0.018	0.003	−6.561	** < 0.001**	−0.019	0.002	−7.773	** < 0.001**
Volcanic/Limestone pre-Quaternary	−0.002	0.003	−0.64	0.523	−0.008	0.002	−3.153	**0.002**
**Reach scale**								
Reach elevation	−0.044	0.006	−7.483	** < 0.001**	−0.022	0.006	−3.348	**0.001**
Riparian forest	−0.013	0.004	−3.64	** < 0.001**	−0.006	0.004	−1.744	0.084
**Random Effects**								
Residual standard deviation (σ)	1.518				0.826			
Site (intercept) standard deviation (τ_00_)	1.081				1.323			
Site (AT) standard deviation (τ_11_)	0.09				0.135			
Correlation (WT) at lag 1 (*ρ*)	0.962				−			
Intraclass correlation (ICC)	0.33				0.7			
Observations	38,667				1,193			
Marginal /Conditional R^2^	0.719/0.805				0.775/0.931			

Significant values are in bold.

**Figure 2:**
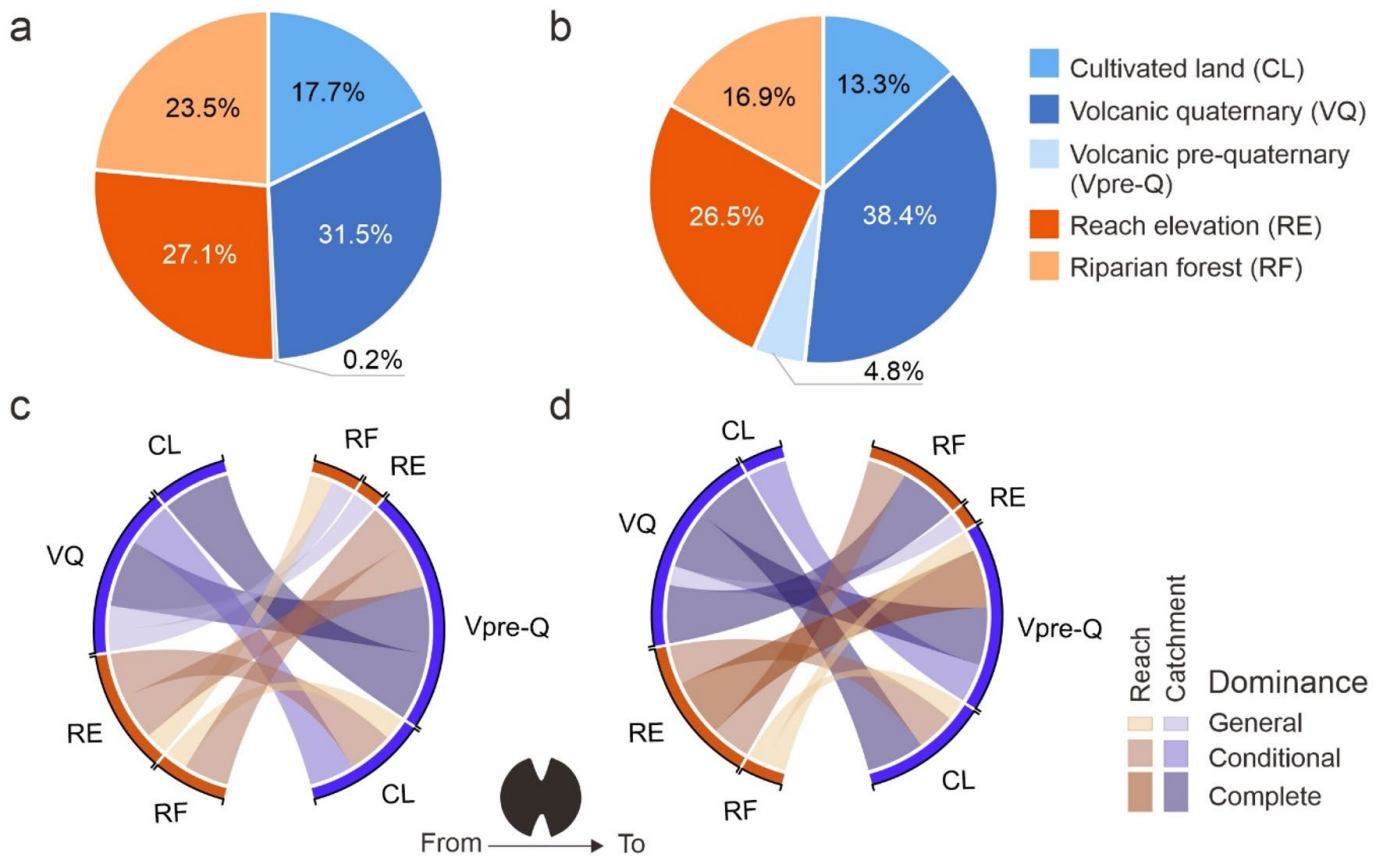
Relative importance and pair-wise dominance analysis among environmental covariates. (**a**-**b**) Relative importance for each environmental covariate in the (**a**) daily and (**b**) monthly regression models measured using the marginal coefficient of determination R^2^ and expressed as their overall average contribution towards the observed variance in water temperatures explained by the full model after accounting for the effect of air temperature (baseline model). (**c**-**d**) Chord diagrams showing the pairwise dominance relationships between covariates in terms of their relative contribution towards explained variance across all possible subset models for the (**c**) daily and (**d**) monthly models. Dominance is established from the left side predictors towards the right side predictors in the diagrams; the width and colour of the link indicating the dominance level. Three hierarchical levels of dominance are established according to the strength of the control of one variable over the other, which are in decreasing order: complete dominance (i.e., for all individual subset models), conditional dominance (i.e., averaged over subset models for each model size), and general dominance (i.e., averaged across all subset models) (see Methods for details). Covariates are colour-coded according to their spatial context at the reach (brown) and catchment (blue) scales. Chord diagrams created with the 'circlize' R package ^42^.

Some environmental predictors had consistent significant effects on water temperature irrespective of the timescale of the temperature data (Table 2). Increasing proportion of quaternary volcanic rocks within the contributing catchment and higher reach elevation had a highly significant negative effect on water temperatures while increasing proportion of cultivated land in the catchment had a significant positive effect. The former two variables had also the highest importance in terms of relative contribution to the variance explained by the fixed-effect component of the model relative to the baseline (Fig. 2a-b), with the proportion of quaternary volcanic rocks accounting for a 31.48% and 38.41%, and reach elevation a 27.06% and 26.53% of the total variance in the daily and monthly models.

Other predictors, however, showed clear differences in their effects and relative importance between the two models (Table 2, Fig. 2a-b). At the reach scale, the cooling effect of riparian cover became weaker with increasing timescale, its effect size halved and shifting from highly significant to marginally non-significant in the monthly model (Table 2); its relative importance reduced from 23.52 to 16.93% (Fig. 2a-b). On the other hand, at the catchment scale, increasing proportion of pre-quaternary extrusive volcanic formations had a significant negative effect on monthly water temperatures that was absent from the daily model (Table 2), while the relative importance of pre-quaternary and quaternary volcanic rocks increased by 4.65%, and 6.9% in the monthly relative to the daily model (Fig. 2a-b).

These results were further supported by dominance analysis (Fig. 2c-d). Despite being the most dominant predictor in both models, the proportion of quaternary volcanic rocks experienced a strong weakening of dominance links on other predictors between timescales, changing from exerting complete dominance over all other predictors but reach elevation in the monthly model to having only general dominance (i.e., lowest hierarchical level of dominance) over riparian forest cover and conditional dominance over the proportion of catchment cultivated land in the daily model. On the other hand, riparian forest cover and cultivated land use increased both one level in dominance hierarchy over the proportion of pre-quaternary volcanic rocks in the daily relative to the monthly model (Fig. 2c-d).

Together, notwithstanding the fact that reach- and catchment-scale covariates remained important at both temporal resolutions, these results point towards a clear temporal dependency of the relative importance and dominance of environmental controls on stream water temperature. The stronger effects, higher relative importance and dominance exerted by reach-scale predictors related to controls governing incident solar radiation and local heat exchange processes at the stream surface observed at the daily timescale became dampened at the monthly timescale by predictors related to catchment-scale hydrological and hydrogeological processes controlling the partitioning, storage and routing of water into the streams. These results hint at potential interdependencies between temporal and spatial scales that will be explored in more detail in future studies.

Cooler daily summer stream temperatures are often associated to the effect of riparian canopy limiting incoming radiation at the water surface resulting in a significant reduction of the net surface heat flux compared to open or non-vegetated riparian zones^43–45^. Similar effects have also been reported, albeit less frequently, in studies focusing on coarser timescales (i.e., monthly or annual temperatures)^46^, perhaps because at these temporal resolutions the interest is often on larger spatial scales where the focus is on predictors related to catchment-scale processes such as land use cover^47^. In any case, our results suggest that riparian cover exerts a larger relative control on water temperature at finer than coarser timescales. Riparian vegetation can influence short-term (e.g., daily) variation in water temperatures via lateral flow of surface and ground water into the stream channel through interception, infiltration and soil saturation processes, particularly in small, steep headwater streams^48,49^.

The relative importance and dominance niche left by riparian cover in the monthly model was filled by the proportion of solid volcanic geology in the contributing catchment. Although the location, direction and rate of groundwater-surface water interactions are controlled by multiple processes that operate over different spatial and temporal scales^50^, basic information on solid geology at catchment level can be an effective proxy for groundwater-streambed interactions^51^. Together with the type of formation, geological age is important as it relates to the processes of weathering, soil development and formation of secondary porosity in underlying rocks, all of which are associated to aquifer recharge and surface–groundwater dynamics^20^. Young extrusive volcanic formations often have high porosity and permeability offering deep, stable aquifers. In our study catchments, young volcanic formations comprised primarily mafic basaltic and andesite rocks, both formations capable of forming prolific unconfined aquifers in which groundwater flow freely through vesicular and fracture systems^20^. On the other hand, older extrusive volcanic rocks often experience a decreasing trend in porosity and hydraulic conductivity with increasing geological age resulting from the filling of vesicles and fractures by secondary material. In such settings, groundwater is largely stored in less stable, shallow aquifers with short flow paths to streams. A study conducted at 70 gauging stations across Japan, including sites located in three of our four study catchments, found a strong correlation between the type and age of dominant catchment geology and base flows with catchments dominated by quaternary volcanic formations supporting the most abundant flows whereas those under older volcanic formations yielded intermediate flows^21^. Shorter residence times and shallower storage also mean warmer groundwater temperature and greater sensitivity to seasonal and long-term changes in air temperatures^52^. The observed weaker but significant negative effect of old volcanic rocks on monthly water temperatures likely reflects to some extent these contrasting conditions.

Catchment cultivated land use had a consistent and significant warming effect on water temperatures irrespective of resolution and, together with riparian forest cover, increased in relative importance and dominance in the daily model. Agricultural land use is often associated to increased evapotranspiration and surface runoff and decreased base flow and water yield^53^; all factors that can contribute towards warming of thermal regimes in rivers particularly during dry seasons^54,55^. Decreased time lag responses between precipitation and streamflow associated to agricultural use^56^ may have contributed towards the observed higher signature of this predictor on mean daily relative to monthly water temperatures.

## Conclusions

Our perception of nature, intrinsically dynamic in time and space, is inevitably conditioned by the scales and degree of detail we observed it with. Paraphrasing Simon A. Levin “there is no single natural scale at which ecological phenomena should be studied; systems generally show characteristic variability on a range of spatial, temporal, and organizational scales”^57^. To understand ecological patterns, and the processes that produce them, it is necessary to analyze and compare them across multiple spatial and temporal scales. The role of scale in defining patterns and processes is especially important to landscape ecology because landscapes comprise many heterogeneous components that interact and show dependence across spatial and temporal scales^58,59^.

Previous studies have documented complex temporal and spatial dependencies in the contribution of climatological, topographical and geological controls to hydrological and hydrochemical responses of streams^14,60,61^. For example, Karlsen et al^62^ found that catchment soil characteristics correlated significantly with specific discharge in a boreal catchment at shorter timescales (monthly to daily), but not when discharge was aggregated into annual timescales. In our study, although reach- and catchment-scale covariates remained important in both models, we found a clear signal for the strong control exerted by the riparian forest cover on daily water temperatures to become dampened by the effect of predictors related to catchment geology in the monthly model. Previous studies have documented the strong, direct control exerted by the type and extent of riparian cover on summer stream temperature variability over short (hour to daily) temporal scales through their influence on the amount of incoming solar radiation received at the stream surface^44^. Increasing the time span over which temperatures are averaged will effectively reduce this variance. This could reduce the effect size, importance and dominance of riparian cover in explaining differences in temperatures across sites between the two models relative to other covariates. On the other hand, groundwater-dominated streams, such as those found in catchments dominated by extrusive volcanic formations in our study, are characterized by cooler, stable summer thermal regimes which signature should be little influenced by the effect of averaging temperatures over increasing time scales. This could explain the observed time-dependent trade-offs between the two covariates in terms of their relative contribution towards explained variance in the models.

Climate change is altering the abundance, distribution and phenology of species across land and oceans, reshuffling biodiversity on a global scale, and modifying ecosystems with dire implications to human well-being^63,64^. Species may counter the effects of climate change through phenotypic and evolutionary adaptation although evidence suggest such responses are often outpaced and imperfect^65^. Freshwater ecosystems are at the frontline of these changes because the linear, dendritic nature of hydrological networks constrain biota responses and concentrate compounded impacts from climate change and the many human activities associated to the use of water as a resource^66^. Robust prediction of water temperatures at reach scale across river networks is therefore necessary for the development of sound river management practices that are resilient and adaptive to the effects of future climate change. Statistical regression models that incorporate heat exchange processes in rivers through the incorporation of relevant environmental proxies represent a useful tool to achieve this goal in a simple, flexible way^6^. Our results highlight the importance of contextualizing such relationships to the temporal resolution of the temperature data informing the models.

## Supplementary Information

Supplementary Information.

## Data Availability

All data sets used for generating the environmental predictors and hydrological networks are publicly available as referenced in Table 1. Air and water temperature data generated by this study is available from the corresponding author on reasonable request.
