# Supplementary material for: Timescale mediates the effects of environmental controls on water temperature in mid- to low-order streams
Source: Sci Rep. 2022 Jul 18;12:12248. doi: 10.1038/s41598-022-16318-9 (PMC9293926; doi:10.1038/s41598-022-16318-9)
Supplement: Supplementary file 1 — Supplementary Information. [file 41598_2022_16318_MOESM1_ESM.docx]

**Supplementary Figures**

**Timescale mediates the effects of environmental controls on air-water temperature relationships in mid- to low-order streams.**

Jorge García Molinos^1*^, Ishiyama Nobuo^2^, Masanao Sueyoshi^3^, Futoshi Nakamura^4^

^1^ Arctic Research Center, Hokkaido University, Sapporo, Japan. ORCID: 0000-0001-7516-1835

^2^ Forestry Research Institute, Hokkaido Research Organization, Bibai, Japan.

^3^ Aqua Restoration Research Center, Public Works Research Institute, Gifu, Japan. ORCID: 0000-0002-5517-0256

^4^ Research Faculty of Agriculture, Hokkaido University, Sapporo, Japan. ORCID: 0000-0003-4351-2578

^*^ Corresponding author: [jorgegmolinos@arc.hokudai.ac.jp](mailto:jorgegmolinos@arc.hokudai.ac.jp) +84-11-706-9628

Submitted to Scientific Reports


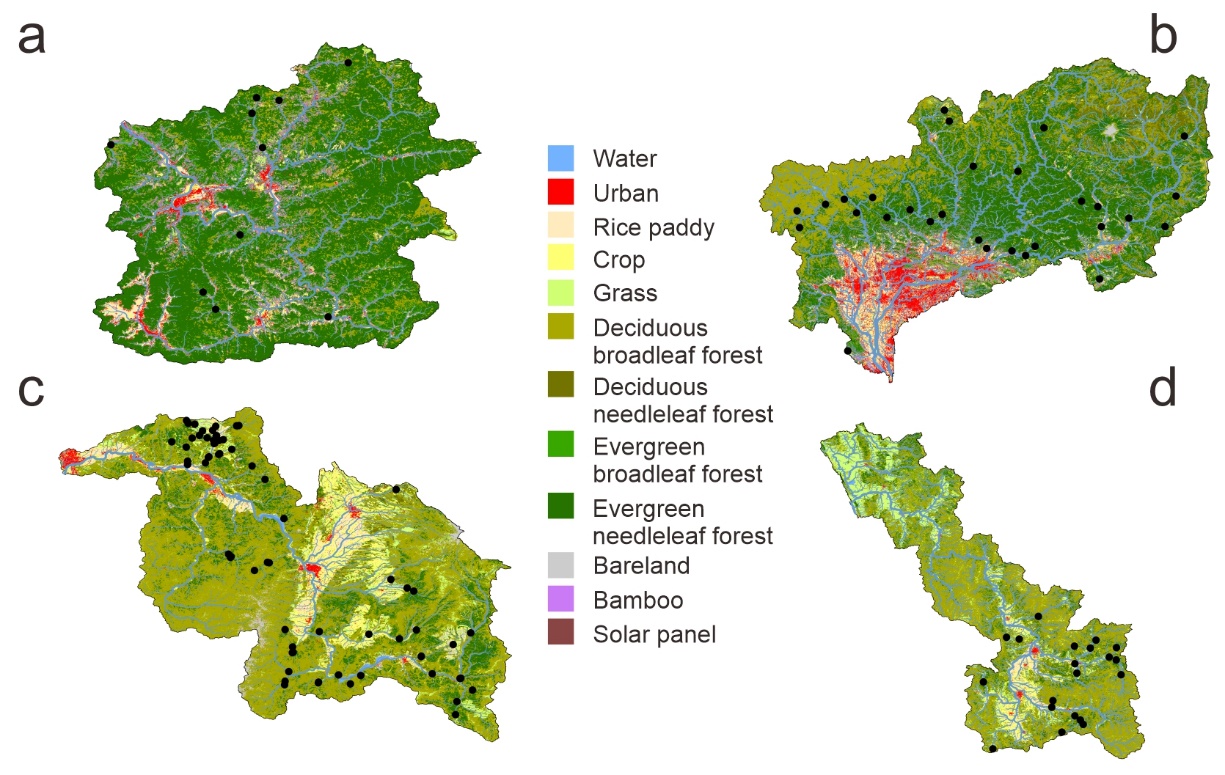


**Fig. S1**. Land use maps for the study catchments: (a) Hiji, (b) Kiso, (c) Sorachi, and (d) Tehsio. Land use classification based on the ALOS / AVNIR-2 High Resolution Land Use/Cover map (2018-2020), Japan Aerospace Exploration Agency (JAXA). Black points correspond to the location of the monitored sites. Maps created with ArcGIS Desktop 10.7.1 (https://www.esri.com).


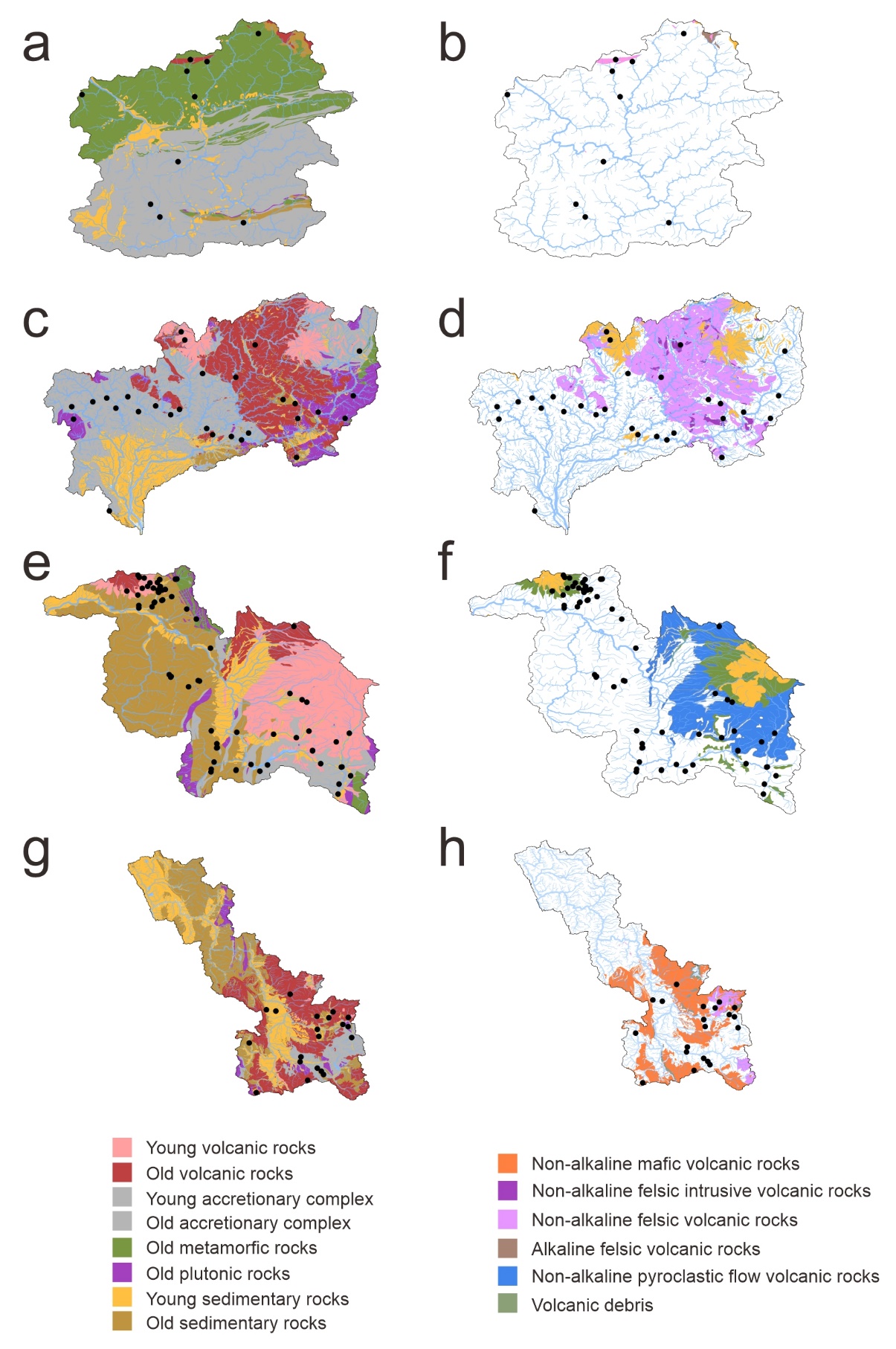


**Fig. S2**. Geology of the study catchments: (a-b) Hiji, (c-d) Kiso, (e-f) Sorachi, and (g-h) Tehsio, grouped as (a, c, e, g) broad geological categories divided by age (old: pre-Quaternary, young: Quaternary), and (b, d, f, h) specific volcanic rock formations. Source: Seamless Digital Geological Map of Japan, Geological Survey of Japan. Maps created with ArcGIS Desktop 10.7.1 (https://www.esri.com).


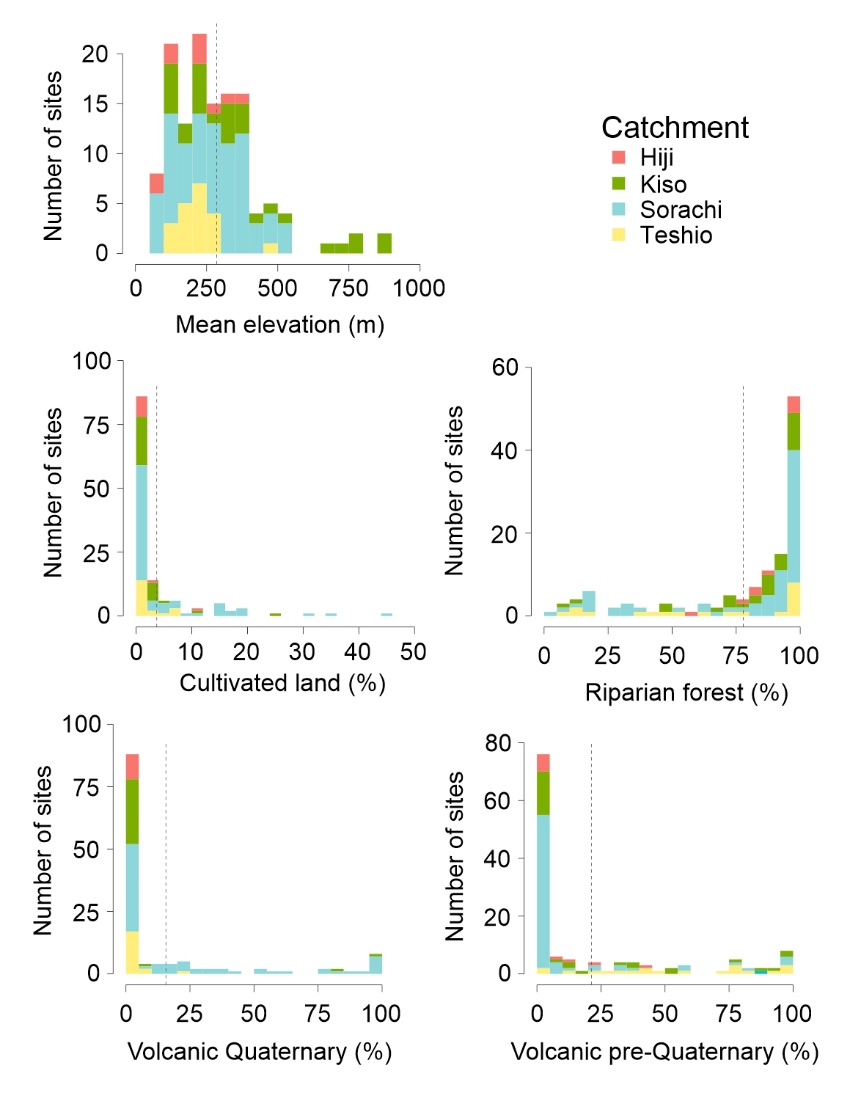


**Fig. S3**. Stacked bar plots showing the distribution of each environmental predictors at each site by catchment. The cultivated land and riparian forest cover categories include respectively the cultivated crop and rice paddy classes and the evergreen, deciduous and bamboo forest classes (see Fig. S1). Vertical dotted line marks the mean value across all monitored sites for each predictor variable.


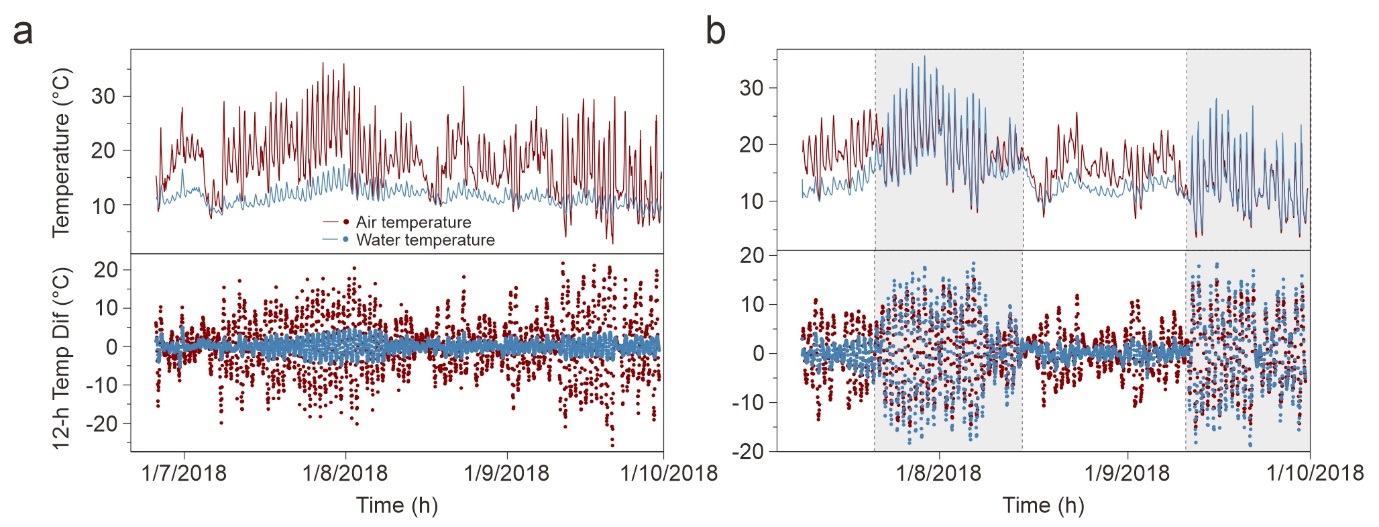


**Fig. S4**. Extracts from the temperature series of two monitored sites exemplifying the visual inspection of potential out-of-water events. The first site (a) shows no unusual behaviour of the water temperature series. The second site (b) shows two suspected out-of-water events (shaded areas) detected as periods where the variability in water temperatures converged with that of air temperatures.

**
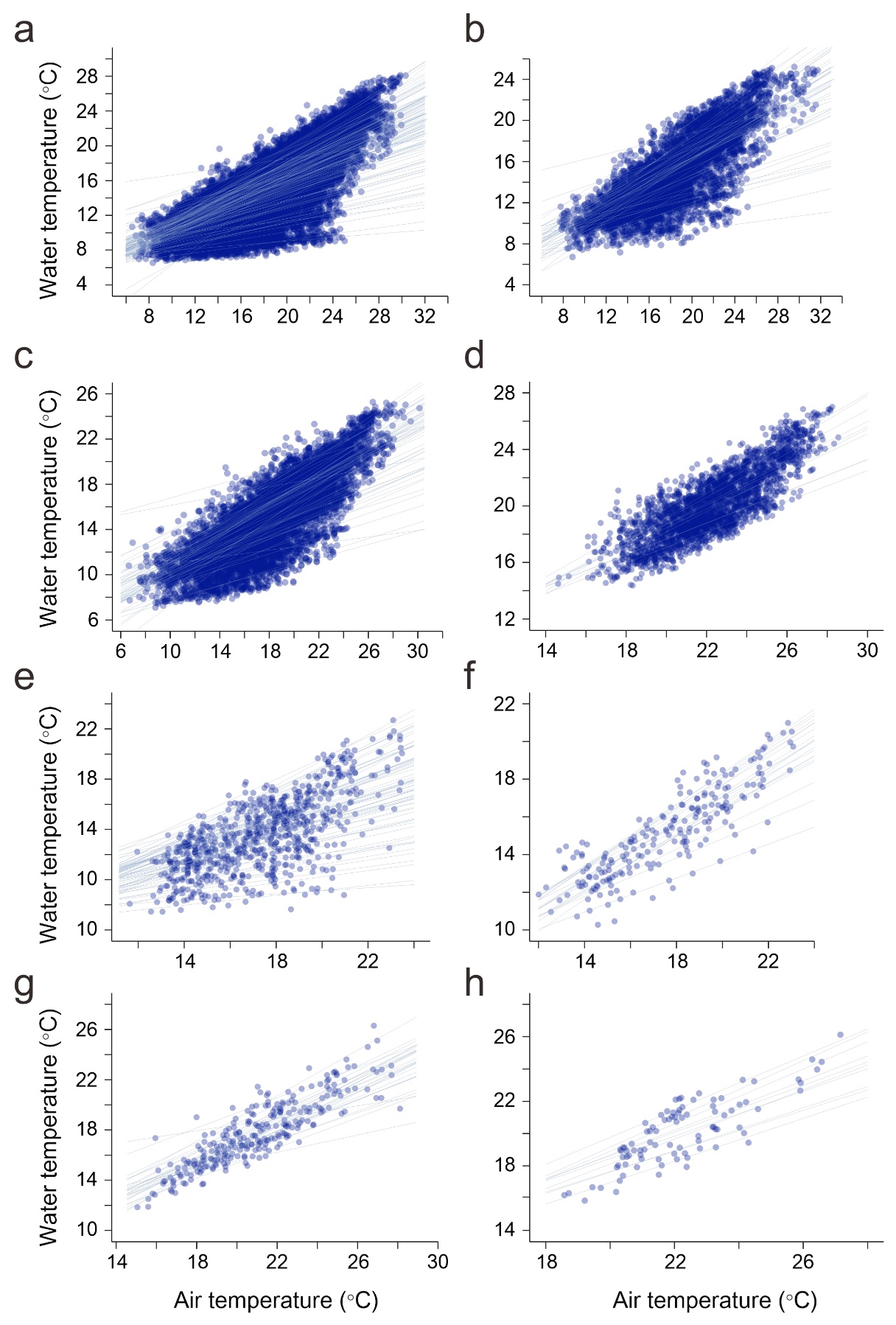
**

**Fig. S5**. Scatterplots of observed (a-d) daily and (e-h) monthly average air and water temperatures across all 130 monitored sites for the (a, e) Sorachi, (b, f) Teshio, (c, g) Kiso, and (d, h) Hiji river basins. Regression lines between air and water temperatures by site across catchments are provided as a reference.

**
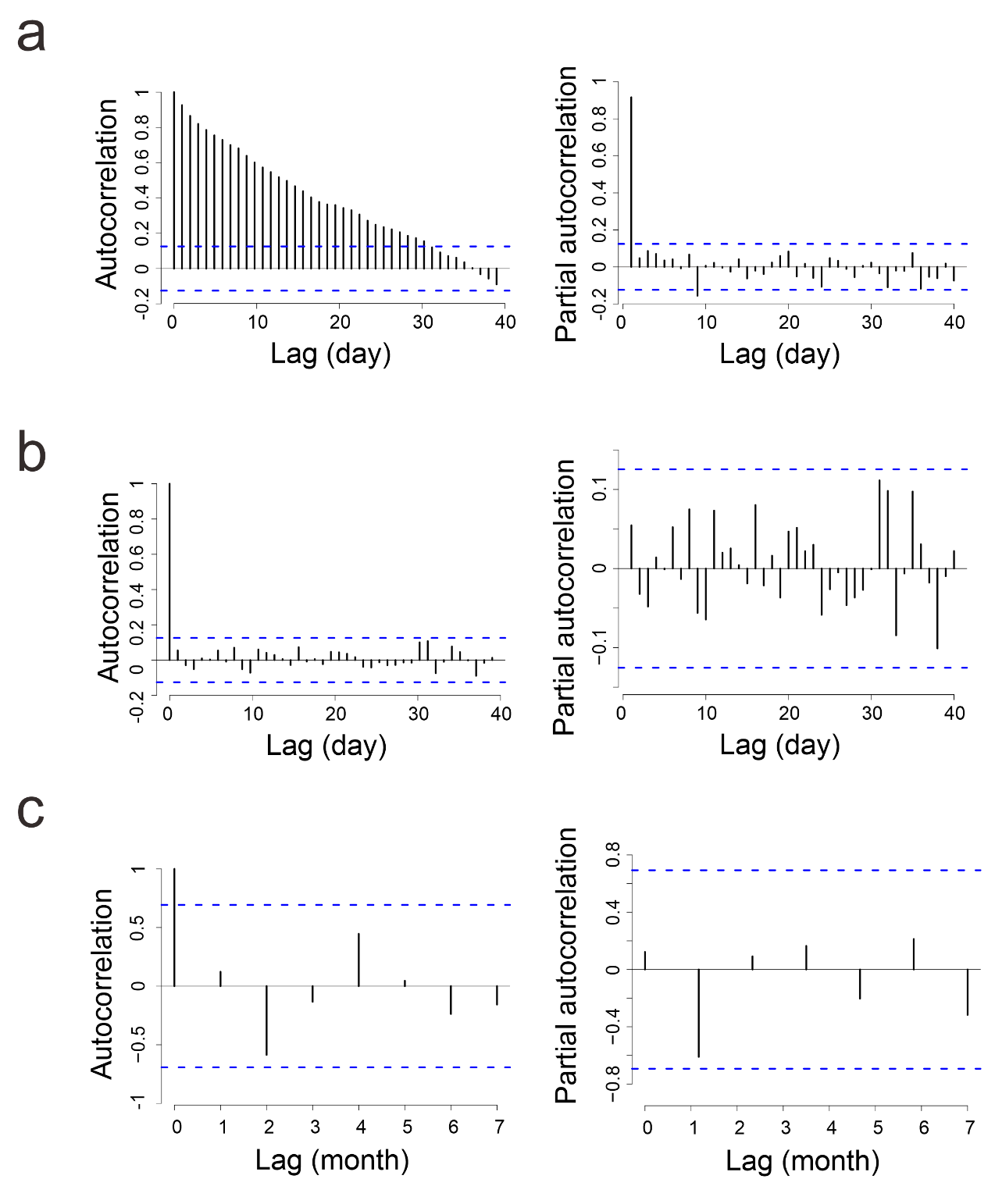
**

**Fig. S6**. Example of the residual autocorrelation and partial autocorrelation function from the (a-b) daily, and (c) monthly LMMs fitting water temperatures as a function of ait temperatures and the suite of reach- and catchment-scale environmental covariates. (a) The residuals from the daily model presented a clear, strong autocorrelation at lag 1, as indicated by the gradually decaying autocorrelation and the single, very strong partial autocorrelation at day/week 1. (b) Inclusion in the model of a continuous autoregressive error structure of lag 1 (corCAR(1)) solved this issue. (c) Residuals from the monthly model did not present temporal correlation patterns across sites.
